# Endoscopic Removal of Impacted Barium Stools Using a Snare and a Long Hood: A Rare Complication Following Upper Gastrointestinal Barium X‐ray Radiography (With Video)

**DOI:** 10.1002/deo2.70182

**Published:** 2025-08-06

**Authors:** Takaaki Kishino, Naoki Okamoto, Kohei Fukumoto

**Affiliations:** ^1^ Department of Gastroenterology and Hepatology Center for Digestive and Liver Diseases Nara City Hospital Nara Japan; ^2^ Department of Gastroenterology and Hepatology Saiseikai Suita Hospital Osaka Japan

**Keywords:** barium sulfate, complication, endoscopic surgical procedure, fecal impaction, upper gastrointestinal series

## Abstract

Barium sulfate is widely used as a radiographic contrast medium in upper gastrointestinal barium X‐ray radiography for cancer screening in Japan. Although generally considered safe, rare complications such as bowel obstruction due to impacted barium stools can occur. We report the case of a healthy 41‐year‐old woman who developed colonic obstruction following upper gastrointestinal barium X‐ray radiography. Endoscopic treatment was attempted using a snare and a long hood to fragment and remove the barium mass. The impacted stools were successfully removed endoscopically, and there were no serious complications such as bowel perforation or generalized peritonitis that would require surgery. This case demonstrates that endoscopic removal can be an effective and less invasive treatment option for barium impaction, which carries a risk of requiring surgical intervention. Although patients are usually advised to hydrate and take laxatives after the examination, this case highlights the importance of ensuring that patients fully understand the potential risk and the need to seek prompt medical attention.

## 1 Introduction

Barium sulfate is a commonly used radiographic contrast medium in examinations for gastrointestinal diseases, including in population‐based gastric cancer screening programs in Japan [1]. The incidence of complications in a barium study is low [1, 2], but rare complications have been reported, including intestinal obstruction [3, 4, 5, 6, 7, 8] and subsequent perforation [3, 4, 5] caused by impaction of barium stools (barolith). The Japanese Society of Gastrointestinal Cancer Screening (JSGCS) has conducted a national survey on complications associated with upper gastrointestinal barium X‐ray radiography screening. In the 2021 survey, bowel obstruction was reported in three cases out of 3,077,580 screenings, and intestinal perforation in two cases [9].

A barolith is a dense, hard mass formed from inspissated barium mixed with feces and can lead to intestinal obstruction [7]. When conservative management fails, surgical intervention is often necessary [2, 3, 6, 8, 10], and there are reports of fatal complications such as intestinal perforation [3]. However, endoscopic management is not well established, and reports in the literature remain scarce. Here, we present a case in which impacted barium stools were endoscopically removed in a healthy woman, thereby avoiding surgical intervention.

## 2 Case Report

A healthy 41‐year‐old woman underwent an upper gastrointestinal barium X‐ray radiography as part of routine cancer screening. She had a past medical history of open abdominal surgery for appendicitis at age 6 and a Cesarean section at age 28. Other than these, she had no significant medical history and no tendency toward constipation. She passed a small amount of stool the day after the procedure, but subsequently developed constipation. Seven days later, she presented to our hospital with severe lower abdominal pain. On admission, her vital signs were as follows: body temperature, 35.8°C; blood pressure, 112/53 mmHg; and pulse rate, 64 bpm. Physical examination revealed abdominal distension and tenderness without peritoneal signs. Surgical scars were observed in the right lower abdomen and lower midline region. Blood test results on admission revealed mild anemia, with a hemoglobin level of 10.6 g/dL and a hematocrit of 33.9%. However, inflammatory markers were not elevated; the white blood cell count was 8.21×10^9^/L, and C‐reactive protein was 0.01 mg/dL. No other significant abnormalities were noted in the remaining laboratory parameters (Table S1).

**FIGURE 1:**
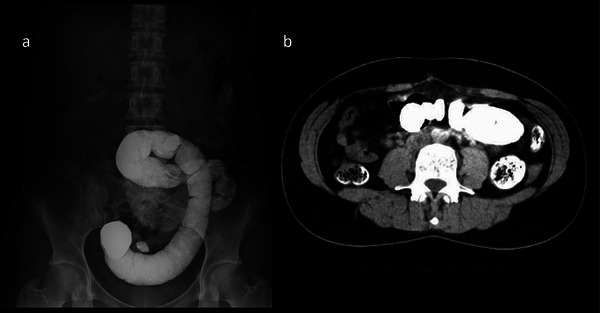
Abdominal X‐ray and contrast‐enhanced computed tomography revealed a large amount of retained barium in the sigmoid colon without evidence of free air. (a) Abdominal X‐ray, (b) Abdominal computed tomography.

Abdominal X‐ray and contrast‐enhanced computed tomography revealed a large amount of retained barium in the sigmoid colon with no evidence of free air (Figure 1). Due to the risk of perforation or generalized peritonitis from increasing intraluminal pressure, we performed an urgent colonoscopy to remove the barium impaction without prior bowel preparation.

**FIGURE 2:**
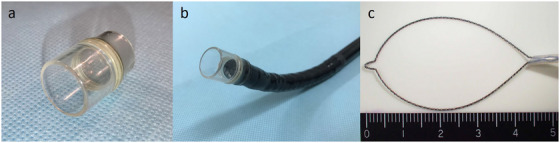
(a) A long hood (distal attachment, MAJ‐663; Olympus). (b) A water‐jet scope (PCF‐Q260AZI; Olympus) with the long hood. (c) A snare with a loop diameter of 25 mm (Snare Master [SD‐210U‐25]; Olympus).

The colonoscopy was performed using a water‐jet colonoscope (PCF‐Q260AZI; Olympus) fitted with a long hood (MAJ‐663, Olympus; outer diameter 15.8 mm) (Figure 2a,b). The barium stools were found to be impacted at a sharp bend in the sigmoid colon. We used a 25‐mm snare (Snare Master [SD‐210U‐25], Olympus; Figure 2c) to mechanically fragment the mass, which was then suctioned into the hood cup and removed (Video S1). The long hood, which has no side holes, allowed for effective suction of the barium debris with strong negative pressure.

**FIGURE 3:**
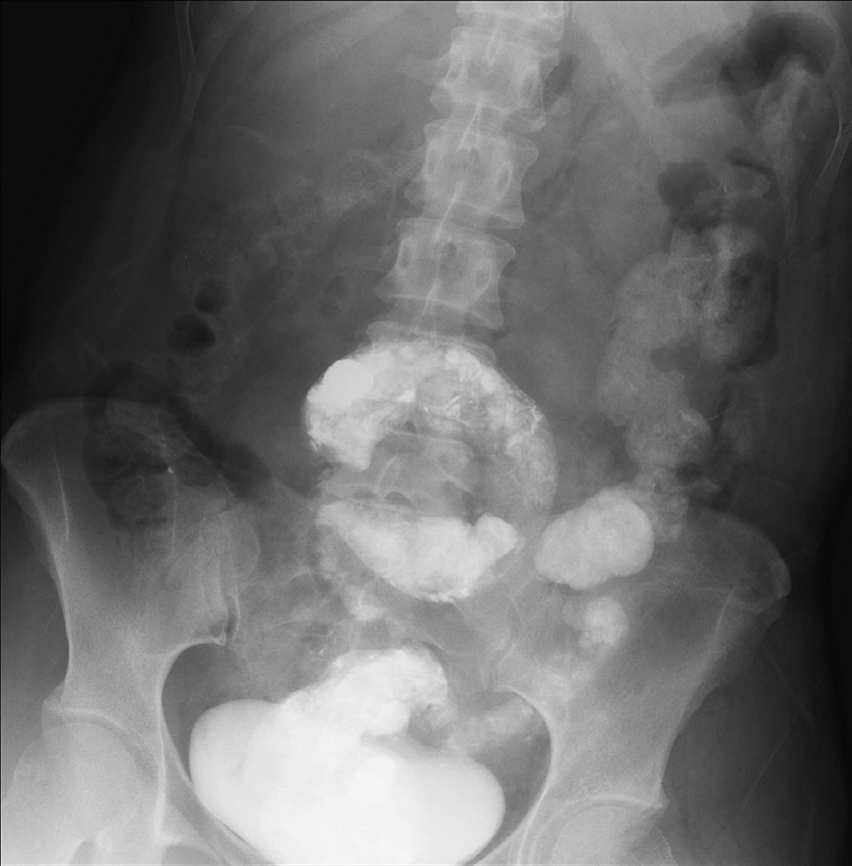
Abdominal X‐ray after treatment.

By repeatedly fragmenting and suctioning the stools, most of the impaction was cleared in approximately 60 min. Endoscopic observation confirmed that the obstruction was resolved, and the lumen was adequately decompressed, although some residual barium remained (Figure 3). It also revealed no significant bowel narrowing or stiffness in the sigmoid colon, suggestive of adhesions. The patient's abdominal pain improved immediately post‐procedure. However, she developed localized peritonitis in the left lower quadrant the next day, characterized by tenderness with localized peritoneal signs and elevated inflammatory markers in blood tests (white blood cell count: 18.08 × 10^9^/L and C‐reactive protein: 10.2 mg/dL) (Table S1). Abdominal X‐ray showed no free air. Based on the clinical course, we diagnosed localized peritonitis secondary to ischemic colitis due to bowel obstruction. She was managed conservatively with antibiotics. She recovered without further complications and was discharged on day 5 of hospitalization.

## 3 Discussion

Barium‐induced colonic obstruction is rare [2]. In a systematic review conducted by Kurer et al., only 31 patients with barolith impaction were identified between 1950 and 2006. According to this review, in cases where the site of obstruction was specified, more than half occurred in the left colon (i.e., the descending or sigmoid colon). This may be attributed to the narrowing of the colonic lumen as it progresses distally, allowing baroliths to accumulate as barium mixes with intestinal contents. Barium‐induced colonic obstruction is usually reported in elderly or bedridden patients with underlying bowel motility disorders [2, 8]. However, this case demonstrates that this complication can occur even in healthy individuals with no history of constipation but a history of open abdominal surgery.

For treatment, conservative management such as fasting, laxatives, or enemas is generally the first‐line approach [7]. If ineffective, or if complications such as perforation or generalized peritonitis are present, surgical interventions such as colectomy or colostomy are considered [3, 4, 5]. According to the aforementioned systematic review, approximately one‐third of the reported cases (11/31) were successfully managed with conservative measures, including the use of laxatives. Notably, surgery was required in nearly half of the patients (14/31). Even more interestingly, endoscopic dissolution was attempted in only three cases. Based on these findings, we conducted a review of case reports describing endoscopic treatment for barium‐induced colonic obstruction (Table 1). Including the present case, a total of nine cases of endoscopic treatment have been reported, with the site of obstruction located in the left colon in all cases. Among the nine cases, endoscopic removal was successful in five, while three of the four unsuccessful cases required surgical intervention. These findings suggest that endoscopic treatment is technically challenging and carries a high risk of requiring surgery. Moreover, since all successful cases showed no signs of peritoneal irritation, endoscopic treatment should be limited to patients with stable vital signs and no clinical or radiological evidence of generalized peritonitis or perforation. In this case, given the severity of symptoms and the risk of progression to perforation or generalized peritonitis, we determined that early endoscopic decompression was necessary. Although the patient developed localized peritonitis secondary to ischemic colitis due to bowel obstruction after the endoscopic procedure, her abdominal pain improved, and surgical intervention was avoided. Therefore, timely endoscopic intervention was considered a valuable treatment option for avoiding surgical intervention.

**TABLE 1:** Review of case reports on endoscopic treatment for barium‐induced intestinal obstruction.

Reference	Year	Sex	Age	Comorbidities/Medical history	Time from barium study to onset	Clinical presentation	Site of obstruction	Endoscopic devices used	Endoscopic outcome	Additional treatment	Clinical outcome
Cheney [1]	1994	Male	69	End‐stage renal disease on dialysis	3 days	Abdominal pain with distension and vomiting	Descending colon	Water irrigation and snare	Unsuccessful	Water irrigation via a decompression tube	Recovery
McDonnell [2]	1997	Female	54	Lung cancer; Billroth II gastric resection	10 days	Diffuse abdominal tenderness, but no peritoneal signs	Sigmoid colon	Not recorded	Unsuccessful	Sigmoid colectomy with a Hartmann's procedure for perforation	Recovery
Kurer [3]	2007	Female	64	Ulcerative colitis	9 months	Abdominal pain	Descending colon	Water‐jet	Successful	None	Recovery
Thosani [4]	2014	Female	39	Scleroderma	7 days	Nausea, vomiting, abdominal pain, and obstipation	Sigmoid colon	Water‐jet and biopsy forceps	Successful	None	Recovery
Shaughnessy [5]	2015	Female	86	Billroth II gastric resection	1 week	Diffuse abdominal pain and distension; septic shock	Descending colon	Not recorded	Unsuccessful	Total colectomy with end‐ileostomy	Recovery
Iida [6]	2017	Female	45	Chronic constipation	7 days	Lower abdominal pain without peritoneal signs	Sigmoid colon	Water‐jet, net, and snare	Successful	None	Recovery
Vieiro [7]	2023	Female	62	Not recorded	Not recorded	Abdominal pain, nausea, and constipation	Descending colon	Water irrigation and snare	Successful	None	Recovery
Sharpe [8]	2024	Female	67	Diabetes mellitus; Schizoaffective disorder	Not recorded	Diffuse abdominal tenderness with guarding	Sigmoid colon	Not recorded	Unsuccessful	Left colectomy with Hartmann's procedure for sigmoid colon perforation	Recovery
This case	2025	Female	41	Open appendectomy; Cesarean section	7 days	Severe lower abdominal pain; tenderness without peritonitis	Sigmoid colon	Water‐jet, long hood, and snare	Successful	None	Recovery

*Note*: We searched for published articles indexed in PubMed up to June 2025 using the following search terms: (“barium”[Title/Abstract] OR “barolith”[Title/Abstract] OR “bariolith”[Title/Abstract] OR “barium sulfate”[MeSH Terms]) AND (“obstruction”[Title/Abstract] OR “impaction”[Title/Abstract] OR “ileus”[Title/Abstract] OR “intestinal obstruction”[MeSH Terms]). In addition, we manually reviewed the reference lists of the retrieved articles to identify any further relevant publications. We included case reports that provided detailed clinical information and described endoscopic treatment for barium‐induced intestinal obstruction. The full list of references included in this review is provided in the Supporting Information.

To date, there have been only a few reports on endoscopic treatment for barium impaction [3, 7, 8], and a considerable proportion of these attempts have been unsuccessful [3, 8]. This case report is particularly valuable as it presents a detailed presentation of the endoscopic procedure with video. Because of the high viscosity and density of barium, endoscopic removal is technically challenging. However, the combination of mechanical fragmentation using a snare and strong suction through a long hood without side holes proved effective in this case. We believe that this technique may also be applicable for the endoscopic removal of fecal impaction.

Barium can remain in the colon for several days to months [4], and impaction may occur even in patients with no prior constipation or bowel disease if the elimination of barolith is delayed. Patients are generally advised to hydrate and take laxatives after undergoing barium examinations. They are also instructed to seek medical attention if they do not have sufficient bowel movements. Although this patient had received these instructions, she did not visit a hospital despite having little to no bowel movement during that time, and sought medical attention when severe abdominal pain developed seven days after the examination. Therefore, it is important to ensure patients fully understand the risks and the importance of seeking care for prolonged constipation or abdominal pain [8].

In conclusion, we advocate for accumulating more case reports and procedural experiences to establish effective techniques and indications for endoscopic treatment, as well as appropriate patient management, for such rare complications.

## Ethics Statement

The patient in this case report was treated within the standard scope of care under Japan's national health insurance. Informed consent was obtained from the patient for the medical treatment and procedures described. For the purpose of publication, all accompanying images and videos have been anonymized to ensure that the individual cannot be identified.

## Conflicts of Interest

The authors declare no conflicts of interest.

## Supporting information




**Table S1**: Laboratory data.

**Video S1**: Endoscopic removal of impacted barium stools using a snare and a long hood.
The video shows the process of removing the impacted barium stools endoscopically. The barium stools were impacted at a sharp bend in the sigmoid colon. First, we cut the stools with the snare. The fragmented stools were then suctioned into a long hood cup and removed. By repeating the same procedure, most of the stools were removed in 60 min.

**Supporting File 3**: deo270182‐sup‐0001‐SuppMat.docx
