# Supplementary material for: Endoscopic Removal of Impacted Barium Stools Using a Snare and a Long Hood: A Rare Complication Following Upper Gastrointestinal Barium X‐ray Radiography (With Video)
Source: DEN Open. 2025 Aug 6;6(1):e70182. doi: 10.1002/deo2.70182 (PMC12328090; doi:10.1002/deo2.70182)
Supplement: Supplementary file 1 — Table S1: Laboratory data. [file DEO2-6-e70182-s003.docx]

| **Supplementary Table 1. Laboratory data.** | |  |  |
| --- | --- | --- | --- |
|  | **Day 1**  **(Admission day)** | **Day 2**  **(Post-admission day 1)** | **Reference range** |
| White blood cell count, ×10^9^/L | 8.21 | 18.08 | 3.9–9.8 |
| Neutrophils, % | 69.7 | 85.2 | 40.0–74.0 |
| Red blood cell count, ×10^12^/L | 4.04 | 3.64 | 4.27–5.70 |
| Hematocrit, % | 33.9 | 29.9 | 39.8–51.8 |
| Hemoglobin, g/dL | 10.6 | 9.7 | 13.5–17.6 |
| Platelet count, ×10^10^/L | 33.2 | 32.7 | 13.1–36.2 |
| Total protein, g/dL | 7.2 | 6.2 | 6.7–8.3 |
| Albumin, g/dL | 4.4 | 3.6 | 3.8–5.2 |
| Total bilirubin, mg/dL | 0.38 | 0.64 | 0.3–1.2 |
| Alanine aminotransferase, U/L | 24 | 17 | 5.0–40.0 |
| Aspartate aminotransferase, U/L | 26 | 16 | 10.0–40.0 |
| Lactate dehydrogenase, U/L | 175 | 166 | 124.0-222.0 |
| Alkaline phosphatase, U/L | 59 | 51 | 38.0–113.0 |
| Blood urea nitrogen, mg/dL | 11.5 | 8.3 | 8.0-20.0 |
| Creatinine, mg/dL | 0.73 | 0.72 | 0.65-1.07 |
| Serum sodium, mmol/L | 139 | 137 | 138.0-145.0 |
| Serum potassium, mmol/L | 3.7 | 4.1 | 3.6-4.8 |
| serum chloride, mmol/L | 107 | 106 | 101.0-108.0 |
| C-reactive protein, mg/dL | 0.01 | 10.2 | 0–0.3 |
| Venous blood gas |  |  |  |
| pH | 7.434 | 7.407 |  |
| Partial pressure of CO₂, mmHg | 32.4 | 33.9 |  |
| Partial pressure of O₂, mmHg | 37.5 | 56.6 |  |
| Bicarbonate, mmol/L | 21.2 | 20.9 |  |
| Base Excess, mmol/L | -2.3 | -3.2 |  |
| Lactate, mmol/L | 1.9 | 0.8 | 0.5–2.0 |
