# Supplementary material for: Endoscopic Removal of Impacted Barium Stools Using a Snare and a Long Hood: A Rare Complication Following Upper Gastrointestinal Barium X‐ray Radiography (With Video)
Source: DEN Open. 2025 Aug 6;6(1):e70182. doi: 10.1002/deo2.70182 (PMC12328090; doi:10.1002/deo2.70182)
Supplement: Supplementary file 3 — Supporting File 3: deo270182‐sup‐0001‐SuppMat.docx [file DEO2-6-e70182-s001.docx]

**Reference articles in Table 1**

[1] [Cheney CP, Murphy JR, Wong RK. Colonoscopic dissolution of a barolith. J Clin Gastroenterol 1994;19:265–6.](http://paperpile.com/b/g1Ib49/hWu2)

[2] [McDonnell WM, Jung F. Images in clinical medicine. Barium impaction in the sigmoid colon. N Engl J Med 1997;337:1278.](http://paperpile.com/b/g1Ib49/CxG5)

[3] [Kurer MA, Chintapatla S. Images in clinical medicine. Intestinal obstruction due to inspissated barium. N Engl J Med 2007;356:1656.](http://paperpile.com/b/g1Ib49/0YPu)

[4] [Thosani N, Wolf DS. Images in clinical medicine. Barium concretion causing obstipation. N Engl J Med 2014;371:e8.](http://paperpile.com/b/g1Ib49/xHUd)

[5] [Shaughnessy GF, Cho P, Francis DL. A rare complication of a barium-contrast study. Clin Gastroenterol Hepatol 2015;13:e67–8.](http://paperpile.com/b/g1Ib49/85l0)

[6] [Iida T, Hirano T, Onodera K, Kubo T, Yamashita K, Yamano H, et al. Endoscopic removal of an impacted barolith at the sigmoid colon: a rare case report. Clin J Gastroenterol 2017;10:361–3.](http://paperpile.com/b/g1Ib49/WF4W)

[7] [Vieiro Medina MV, de la Fuente Bartolomé M, García Vásquez C, Jiménez de Los Galanes S. Intestinal obstruction due to bariolith impaction. Rev Esp Enferm Dig 2023;115:519–20.](http://paperpile.com/b/g1Ib49/Unac)

[8] [Sharpe B, Switzer J, Mathews W. Surgical management of barium impaction: A case report. Cureus 2024;16:e61111.](http://paperpile.com/b/g1Ib49/HlpG)
